# Computational models for the classification of antibody specificity using heavy chain features

**DOI:** 10.1371/journal.pone.0349143

**Published:** 2026-05-20

**Authors:** Jia Lin, Jiaqi Chen, Linxuan Wan, Weinan He, Yuxin Zhu, Mu Qiao, Fancun Meng, Di Lin, Yan Che, Zicheng Cao

**Affiliations:** 1 School of Public Health, Shantou University, Shantou, P.R. China; 2 School of Mathematics and Computer Science, Shantou University, Shantou, P.R. China; 3 Local Government Development Research Institute of Shantou University, Law School of Shantou University, Shantou, P.R. China; 4 Engineering Research Center for Big Data Application in Private Health Medicine of Fujian Universities, Putian University, Putian, P.R. China; 5 School of Public Health (Shenzhen), Shenzhen Campus of Sun Yat-sen University, Shenzhen, P.R. China; 6 Shenzhen Key Laboratory of Pathogenic Microbes & Biosafety, Shenzhen Campus of Sun Yat-sen University, Shenzhen, P.R. China; Jinan University College of Pharmacy, CHINA

## Abstract

**Background:**

Antibodies play a critical role in immune defense, with their antigen specificity primarily governed by the unique sequences of their heavy chains, rendering them invaluable tools in research and diagnostics. High-throughput sequencing technologies have facilitated comprehensive profiling of the immune repertoire, generating vast antibody sequence datasets that necessitate advanced analytical methods.

**Methods:**

In this study, we utilized curated antibody sequences from NCBI databases to develop computational classification models for categorizing antibodies into predefined antigen classes. We extracted multifaceted features from the heavy chain sequences, encompassing physicochemical properties, structural composition, sequence order, and evolutionary information. These features were input into machine-learning classifiers to predict antigen specificity across five classes of antibodies: anti-dengue virus, anti-influenza virus, anti-tetanus bacillus, anti-SARS-CoV-2, and anti-Mycobacterium tuberculosis.

**Results:**

Five tree-based machine-learning models were employed, with CatBoost achieving the highest accuracy of 0.7713. To further enhance predictive performance, we developed a stacking model leveraging multiple algorithms, resulting in an improved accuracy of 0.7803. Additionally, a Feature-Based Transformer deep-learning architecture was implemented, yielding an accuracy of 0.7399 and an F1-score of 0.6761. To elucidate the key determinants of antibody-antigen interactions, we applied the SHAP framework to assess feature importance. Among the top 30 contributing features, those representing sequence order and evolutionary information predominated, with amino acids such as cysteine (C), isoleucine (I), histidine (H), and phenylalanine (F) exhibiting notable SHAP values. Notably, cysteine (Cys) emerged as the most influential feature, underscoring its critical role in antibody structure and function. Specific antibodies contributed variably to these key features; for instance, the anti-tuberculosis antibody accounted for approximately 11% of a sequence order feature associated with alanine (A), while the anti-SARS-CoV-2 antibody contributed about 9.26% to a feature associated with isoleucine (I).

**Conclusions:**

Our study demonstrates the efficacy of machine-learning and deep-learning approaches in classifying antibodies into specific antigen categories, providing sequence-based insights into features associated with antibody specificity. These findings have significant implications for the mechanistic understanding, isolation, and development of potential therapeutic antibodies.

## Introduction

The immune system can provide protection against foreign antigens through the production of antibodies, and human antibodies are an important part of the human immune system, which produces a diverse pool of antibodies in response to microbial infections, vaccinations, autoimmune diseases or cancer [1]. The diversity of antibodies is therefore fundamental to the adaptability and effectiveness of the immune system. Analyzing the different sequences in the antibody pool and exploring antigen-antibody interactions [2] can yield important information about diseases, for example, identifying the type of disease by the type of antibody and influencing aspects such as biotherapeutic drugs, immunization and vaccines [3,4].

Recent advancements in high-throughput sequencing technologies have resulted in an unprecedented accumulation of antibody sequence data [5,6]. These vast datasets hold the potential to revolutionize our understanding of antibody specificity, yet their sheer volume and complexity necessitate the development of sophisticated computational models to effectively interpret them. To address these complexities, researchers have increasingly employed machine learning and deep learning methods [7]. Machine learning algorithms have been utilized to classify and identify antigens [8], while deep learning models such as Immune-Builder have been applied to predict the structures and functions of antibodies. Other studies have employed deep learning algorithms, like RPEMHC, to predict the binding affinity between peptides and major histocompatibility complexes (MHCs) [9]. Furthermore, an ensemble learning framework known as iBCE-EL has been developed to enhance linear B-cell epitope prediction, offering improved performance over traditional methods [10]. Despite the progress made with these approaches, traditional bioinformatics tools often fall short in capturing the intricate patterns within the antibody-antigen interaction landscape, highlighting the need for more advanced techniques.

While significant strides have been made in applying AI to antibody research, many existing models focus on paired heavy and light chains, which represent the complete antigen-binding unit. These paired-chain models are undoubtedly the gold standard for late-stage antibody engineering and affinity maturation, as they capture the synergistic interplay between VH and VL domains. However, their application is often limited by the scarcity of large-scale, functionally annotated paired-chain sequence data. In this study, we specifically chose to focus on heavy-chain-only sequences, positioning our approach as a complementary strategy for the early stages of therapeutic antibody discovery. This choice is motivated by the key factors below: Early groundbreaking studies, such as Padlan’s analysis of crystal structures, revealed that specific amino acids like tyrosine and tryptophan exhibit significant enrichment in antigen-binding sites, while the framework region consists of a different set of conserved residues [11]. It is well-established that the heavy chain, particularly the highly diverse complementarity-determining region H3 (CDR-H3), often serves as the primary determinant of antigen specificity and binding energy [12]. This biological principle suggests that a significant portion of the specificity signal is encoded within the heavy chain alone. Also, from a practical standpoint, the vast majority of publicly available antibody sequence data, especially from high-throughput immune repertoire sequencing (Rep-seq), consists of unpaired heavy chains. Developing models that can effectively mine these massive datasets unlocks a rich resource for identifying novel antibody candidates that would otherwise be inaccessible to paired-chain models.

Therefore, our model is not intended to replace paired-chain analysis but to serve as a high-throughput triage tool. It can rapidly screen millions of heavy-chain sequences to prioritize a smaller, more manageable set of promising candidates. These selected heavy chains can then be subjected to further experimental validation, for instance, by pairing them with a universal light chain or a library of diverse light chains to identify functional, high-affinity therapeutic leads. This approach significantly streamlines the initial discovery funnel, making the overall process more efficient.

## Materials and methods

### Dataset

To establish robust models for the classification of antigen-specific antibodies, the construction of a high-quality, non-redundant sequence dataset is crucial. We collected antigen-specific immunoglobulin sequences from the National Center for Biotechnology Information (NCBI) Protein database [13] (https://www.ncbi.nlm.nih.gov/protein/), which contains protein sequences from various biological species, including but not limited to humans, mice, viruses, bacteria, and plants. Antibody sequences were retrieved from the NCBI Protein database. For each antigen category, search strings were constructed in the format: (“anti- [antigen name]” AND “heavy chain”) under the “All Fields” setting. No organism restriction was applied. The amino acid preferences shown are based on the entire VH region, without distinction between framework or CDR regions. The dataset encompassed five classes of antibodies targeting major human infectious diseases: anti-dengue virus, anti-influenza virus, anti-tetanus bacillus, anti-SARS-CoV-2, and anti-Mycobacterium tuberculosis antibodies. Limited by the annotation information of the original data, specific records of dengue virus serotypes (e.g., DENV-1 to DENV-4) were not available, which may have a certain impact on the model’s ability to capture dengue-specific antibody features. We implemented a rigorous preprocessing pipeline to ensure data quality and reduce redundancy, including sequence redundancy reduction, missing data handling, sequence length standardization, and non-standard amino acid handling. Specifically, we utilized CD-HIT [14](Cluster Database at High Identity with Tolerance) to cluster sequences at a 40% identity threshold—a moderate cutoff selected to balance removal of closely related homologs and preservation of functional diversity in antibody VH sequences, consistent with common practices in antibody modeling. Sequences containing substantial missing data were identified during preprocessing. Those with minor gaps were retained, with missing residues temporarily denoted as “X”, whereas sequences with extensive missing regions were excluded to preserve dataset integrity. To ensure uniformity for downstream feature extraction, minimal padding was applied outside terminal regions to align sequences with the median sequence length. Sequences requiring modifications exceeding 10% of their total length were removed, representing less than 1% of the dataset. For compatibility with feature extraction tools such as POSSUM, ambiguous residues were mapped to standard amino acids (e.g., “X” to alanine). Such adjustments affected only a very small fraction of the dataset, and the overall proportion of modified residues relative to the total sequence length was negligible. Therefore, the impact of these preprocessing steps on downstream compositional and evolutionary features is expected to be minimal. A detailed overview of the preprocessing workflow is provided in S1 Fig in S1 File. Following preprocessing, our final dataset comprised 1111 low-redundant antibody sequences across the five antibody classes (Table 1)

**Table 1:** Distribution of antigen-specific antibodies.

Label	Classes	non-redundant sequences
1	Anti-dengue Ab	57
2	Anti-influenza Ab	178
3	Anti-tetanus Ab	84
4	Anti-sars-cov-2 Ab	393
5	Anti-tuberculosis Ab	399

### Feature representation

Based on the preprocessed antibody sequence dataset described above, we constructed a comprehensive feature representation framework to characterize the biological properties of the sequences. Feature extraction is a critical step in developing robust machine learning-based models for protein function prediction [15]. In this study, an 81-dimensional feature vector was generated for each antibody sequence using three complementary encoding strategies: evolutionary information, sequence-order effects, and physicochemical properties. This multi-faceted approach ensures a rich representation of the antibody sequences, capturing both local and global sequence features, with the combined feature set consisting of 20-dimensional AAC-PSSM features for evolutionary information [16], 22-dimensional PseAAC features for physicochemical properties [17,18], and 39-dimensional CTD features for structural and compositional attributes [19].

**Amino Acid Composition – Position-Specific Scoring Matrix (AAC-PSSM)** [16]. To incorporate evolutionary information, we utilized the AAC-PSSM method, which combines amino acid composition with position-specific scoring matrices. PSI–BLAST [17] profiles were generated using the POSSUM web server (https://possum.erc.monash.edu/) [20,21]. For a sequence A of length L, the AAC-PSSM is defined as a 20-dimensional vector:


$$\begin{array}{c} \begin{array}{c} AAC-PSSM(x)=\left(x_{\text{1}},x_{\text{2}},\ldots ,x_{j}{,\ldots ,x}_{\text{20}}\right) \end{array} \end{array}$$ (1)

where $P_{ij}$ is calculated as:


$$\begin{array}{c} \begin{array}{c} x_{j}=\frac{\text{1}}{L}\sum_{i=\text{1}}^{L}\;\;P_{ij} \end{array} \end{array}$$ (2)

Here, $P_{ij}$ represents the PSSM score for amino acid type j at position i. This method effectively captures the propensity for evolutionary conservation or variation at each position in the sequence.

**Pseudo Amino Acid Composition (PseAAC).** To account for sequence-order effects, we employed PseAAC [17,18], which extends traditional amino acid composition by incorporating sequence correlation factors. We utilized the web server at http://www.csbio.sjtu.edu.cn/bioinf/PseAAC/ [22] to generate PseAAC features. The PseAAC vector is represented as:


$$\begin{array}{c} \begin{array}{c} PseAAC=\left[\varphi_{\text{1}},\varphi_{\text{2}},\ldots ,\varphi_{\text{2}\text{0}+\lambda }\right] \end{array} \end{array}$$ (3)

where $\varphi_{i}(i\leq \text{20})$ represents the occurrence frequency of the 20 standard amino acids, and $\varphi_{j}(\text{20}<j\leq \text{2}\text{0}+\lambda )$ represents the *λ* additional factors that incorporate sequence-order information. The weight factor *ω* and correlation factor *λ*λ were optimized to balance the importance of amino acid composition and sequence-order effects.

**Composition, Transition, and Distribution (CTD)**. To capture the global distribution of physicochemical properties along the sequence, we employed the CTD framework [19], focusing specifically on the Composition (C) component. This method categorizes amino acids into three groups based on seven physicochemical properties: hydrophobicity, normalized van der Waals volume, polarity, polarizability, charge, secondary structure, and solvent accessibility. The CTDC (Composition Descriptor) was calculated using the iFeature web server(https://ifeature.erc.monash.edu/) [23], resulting in a 39-dimensional vector representing the frequency of amino acids in each category for each property.

The resulting feature representation integrates complementary aspects of sequence information and serves as input for downstream machine learning models in supervised classification tasks within curated datasets.

### Computational models and evaluation

Leveraging this comprehensive feature set, we implemented a robust computational framework to classify antibody specificity within curated datasets. Our framework incorporates both traditional ensemble methods and deep learning techniques, aiming to capture discriminative patterns associated with specificity labels.

**Fig 1:**
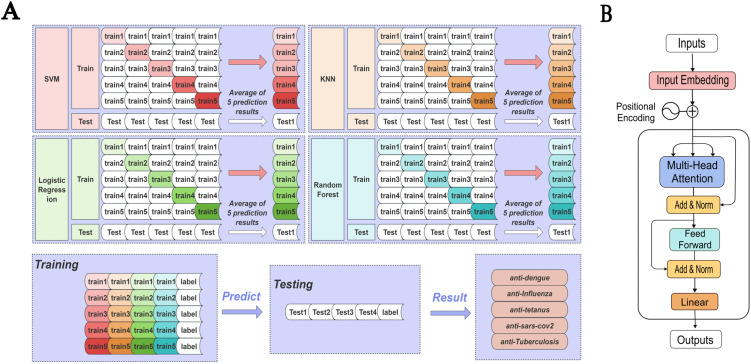
The workflow of the stacking ensemble model and the feature-based Transformer model for antibody specificity prediction. (A) Stacking ensemble framework using five-fold cross-validation. Base classifiers (SVM, Logistic Regression, KNN, and Random Forest) are trained on different folds, and the averaged predictions are used to generate the final classification results for five antibody categories. (B) Architecture of the Transformer model operating on extracted sequence features. The 81-dimensional engineered feature vectors are first projected through an embedding layer with positional encoding, followed by multi-head self-attention and feed-forward layers with residual connections and normalization. A final linear layer produces the classification outputs.

**Ensemble Learning Models**. We developed a two-tiered ensemble learning strategy to improve classification performance. Our approach begins with five robust tree-based algorithms as primary classifiers: Extreme Gradient Boosting (XGBoost) [24], Light Gradient Boosting Machine (LightGBM) [25], Random Forest (RF) [26], Categorical Boosting (CatBoost) [27], and Adaptive Boosting (AdaBoost) [28] -- all configured for native multiclass learning. These models were chosen for their ability to handle high-dimensional data and capture non-linear relationships effectively. Building upon these base models, we implemented a stacking ensemble as a meta-learning approach. This second tier utilizes logistic regression (LR), support vector machine (SVM), and K-Nearest Neighbors (KNN) as base learners, whose outputs are then fed into a Random Forest classifier acting as the meta-learner [29]. The stacking implementation is based on scikit-learn’s StackingClassifier, which internally generates out-of-fold (OOF) predictions via cross-validation to train the meta-learner—ensuring the meta-learner is trained exclusively on OOF predictions and preventing information leakage. This stacking strategy optimizes the integration of base predictions, leveraging the strengths of diverse algorithms to produce more accurate and reliable classification results [30,31]. The detailed framework of our Stacking model is illustrated in Fig 1A.

**Feature-Based Transformer Deep Learning Model.** In addition to ensemble learning methods, we implemented a feature-based Transformer architecture. Unlike sequence-token Transformers that operate directly on raw amino acid sequences, our model applies self-attention to fixed-length, pre-engineered numerical features derived from antibody heavy-chain sequences. The input features are treated as an 81-dimensional embedding representation, which is processed by an eight-head self-attention mechanism to capture global dependencies and interactions among heterogeneous biological descriptors. A 256-dimensional position-wise feedforward network with ReLU activation is subsequently applied to model higher-order feature relationships. To improve robustness and generalization, L2 regularization (λ = 0.56) and dropout (rate = 0.1) were employed. Each of the six stacked encoder layers incorporates residual connections and layer normalization (Fig 1B), ensuring stable training and effective information propagation. This depth was selected to balance model expressiveness with overfitting control under limited data conditions. By modeling feature-level dependencies rather than hierarchical splits, the Transformer provides a complementary perspective to ensemble methods, enabling the detection of subtle global patterns that may be overlooked by tree-based classifiers. Model implementation details and hyperparameters are available on GitHub (https://github.com/LJxp22/AbClass-Classifier).

**Model Training and Evaluation**. The dataset was randomly split into training (80%) and test (20%) sets, maintaining class distribution. All feature data were standardized using z-score normalization. Hyperparameter optimization was performed using Bayesian optimization with 5-fold cross-validation. During training, we fine-tuned the model’s hyperparameters to optimize performance. Additionally, L2 regularization was applied to prevent overfitting and ensure that the model generalizes well to new, unseen data.

For comprehensive performance evaluation, we utilized the following metrics:


$$\begin{array}{c} \begin{array}{c} \begin{array}{cc} & Accuracy=\frac{TP+TN}{TP+TN+FP+FN}\times \text{1}\text{0}\text{0}\% \end{array} \end{array} \end{array}$$ (4)

$$\begin{array}{c} \begin{array}{c} \begin{array}{cc} & Recall=\frac{TP}{TP+FN}\times \text{1}\text{0}\text{0}\% \end{array} \end{array} \end{array}$$ (5)

$$\begin{array}{c} Precision=\frac{TP}{TP+FP}\times \text{1}\text{0}\text{0}\% \end{array}$$ (6)

$$\begin{array}{c} F\text{1}-score=\frac{\text{2}\times Precision\times Recall}{Precision+Recall} \end{array}$$ (7)

where *TP*, *TN*, *FP* and *FN* represent the numbers of true positives, true negatives, false positives and false negatives, respectively.

All performance metrics, including precision, recall, and F1-score, were calculated using weighted averages, which assigns weights to each class based on their sample sizes to ensure the evaluation accounts for class imbalance in the dataset. Additionally, we computed macro-averaged AUROC (Area Under the Receiver Operating Characteristic curve) and AUPRC (Area Under the Precision-Recall curve) to further assess the model’s discriminative power across all classes, providing a robust measure of performance that is less sensitive to class distribution and particularly informative for imbalanced datasets. Visualization techniques, including accuracy and loss curves over training epochs, confusion matrices and ROC curves, were used to assess model performance and classification effectiveness. This framework enables a thorough assessment of classification performance, allowing for meaningful comparisons between approaches and providing insights into their respective strengths and limitations.

### Interpretable framework for quantifying feature impact

To explore the logic driving our classifications and quantitatively describe the impact of specific biological features, we applied an interpretable machine learning framework. We utilized SHAP (SHapley Additive exPlanations) to explain our model outputs by assigning importance values to features. SHAP values are derived from cooperative game theory’s Shapley value, which distributes benefits fairly among participants in a cooperative effort [32]. In the context of machine learning, SHAP calculates the contribution of each feature to the model’s predictions, offering a clear interpretation of the model’s decision-making process.

The SHAP value for a feature is calculated as follows:


$$\begin{array}{c} \begin{array}{c} f\left(x_{ij}\right)=\phi_{\text{0}}(f)+\sum_{j}\;\phi_{ij}\left(f,x_{ij}\right) \end{array} \end{array}$$ (8)

where $x_{i}$ is the ith input sample vector, $f\left(x_{i}\right)$ is the machine learning model’s predicted value for sample $x_{i}$, $\phi_{\text{0}}$ is the based value of the model, and $\phi_{ij}$ is the SHAP value of feature $x_{ij}$. A positive $\phi_{ij}$ indicates that feature $x_{ij}$ contributes positively to the model’s prediction, while a negative $\phi_{ij}\;$indicates a negative contribution. The contribution of each feature was quantified using the following formula:


$$\begin{array}{c} \begin{array}{c} Feature\;Importance=\sum_{i=\text{1}}^{N}\left|\phi_{i}\right| \end{array} \end{array}$$ (9)

where $\phi_{i}$ represents the SHAP value for feature i across all samples N. Features with larger absolute SHAP values were considered more important. The cumulative SHAP values allowed us to rank the features by their contribution to the model’s predictive performance. The integration of SHAP analysis provided a transparent and interpretable view of how specific sequence features influence the prediction of antibody specificity. This approach not only highlighted the key determinants of antibody-antigen recognition but also reinforced the reliability and applicability of our computational models in immunoinformatics [33].

## Results

### Performance of classifiers in antibody specificity classification

Among the evaluated classifiers, the CatBoost model emerged as the best-performing single model. Initially, the model was trained without a validation set, achieving an accuracy of 0.7713, an F1-score of 0.7693, precision of 0.7731, and recall of 0.7713 (Table 2). These metrics reflect the model’s robust and balanced performance in classifying antibodies into predefined specificity categories. Consistent with other performance metrics, Macro-averaged AUROC and AUPRC (S3 Table in S1 File) and corresponding ROC curves (S5 Fig in S1 File) further confirm CatBoost’s superiority. It achieves the highest scores, with a Macro-averaged AUROC of 0.9480 and a Macro-averaged AUPRC of 0.7582, highlighting its ability to mitigate the limitation of class imbalance while maintaining robust multi-class discrimination. In contrast, XGBoost and RF show weaker performance under such imbalance. However, the Stacking model, which combines multiple classifiers—including the four independent models tested prior to its construction (S1 Table in S1 File)—outperformed CatBoost, achieving higher scores across all metrics, with an accuracy of 0.7803, F1-score of 0.7802, precision of 0.7843, and recall of 0.7803. This superior performance suggests that the ensemble method effectively captures more nuanced patterns in the data, offering better generalization across diverse antibody classes. The Feature-Based Transformer, while showing competitive precision at 0.7234, had a lower recall (0.6211), indicating difficulty in identifying all relevant instances, thus underperforming compared to both CatBoost and Stacking in overall classification performance across specificity classes. The best-performing models—CatBoost, Stacking, and Transformer—were further evaluated using confusion matrices on the testing set (S2 Fig in S1 File), offering a clearer view of their classification strengths and limitations. Additionally, all models were trained using 5-fold cross-validation, ensuring robust fitting across the training data (S2 Table in S1 File).

**Table 2:** Performance of various classifiers on the testing dataset.

Classifier	Accuracy	F1-score	Precision	Recall
CatBoost	**0.7713**	**0.7693**	**0.7731**	**0.7713**
XGBoost	0.7578	0.7570	0.7670	0.7578
LGBM	0.7130	0.7196	0.7527	0.7130
AdaBoost	0.7623	0.6317	0.7086	0.6384
RF	0.7534	0.6231	0.7729	0.6508
Transformer	**0.7399**	**0.6761**	**0.7234**	**0.6211**
Stacking	**0.7803**	**0.7802**	**0.7843**	**0.7803**

Performance differences were evident when examining specific antibody classes. For anti-tuberculosis antibodies, all models performed exceptionally well, with CatBoost, Transformer, and Stacking each reaching an accuracy and recall of 0.86 (Table 3). In the classification of anti-SARS-CoV-2 antibodies, Stacking led with an F1-score of 0.85 and precision of 0.90, highlighting its superior capability in distinguishing this class. By contrast, the classification of the anti-dengue antibody class remained challenging for all models, particularly for Stacking, which showed the weakest performance with an accuracy of 0.18 and precision of 0.22. This suggests that features associated with the dengue class are less distinguishable within the dataset, likely due to shared features with other antibodies. Consistent with the testing set, Stacking’s superior performance extended to the training set as well, as indicated by the cross-validated metrics (S2 Table in S1 File). For anti-influenza and anti-tetanus antibodies, moderate success was observed, with Stacking showing better recall (0.75 for anti-tetanus) and precision, demonstrating its adaptability across different antibody types (Table 3).

**Table 3:** Classification performance of different classifiers for antibody specificity on the testing dataset.

Class	Accuracy			F1-score			Precision			Recall		
	C	T	S	C	T	S	C	T	S	C	T	S
Anti-tuberculosis Ab	0.86	0.86	0.86	0.83	0.82	0.84	0.80	0.79	0.82	0.86	0.86	0.86
Anti-SARS-CoV-2 Ab	0.78	0.75	0.81	0.83	0.80	0.85	0.87	0.85	0.90	0.78	0.75	0.81
Anti-dengue Ab	0.27	0.27	0.18	0.33	0.26	0.20	0.43	0.25	0.22	0.27	0.27	0.18
Anti-influenza Ab	0.76	0.82	0.76	0.70	0.78	0.72	0.66	0.74	0.69	0.76	0.82	0.76
Anti-tetanus Ab	0.69	0.50	0.75	0.65	0.52	0.67	0.61	0.53	0.60	0.69	0.50	0.75

### Performance on external validation dataset

To assess the generalizability of our models, we conducted rigorous external validation using an independent dataset from Wang et al. (2022) [20] comprising six classes of antigen-specific antibodies. The external dataset provided only non-redundant immunoglobulin heavy-chain nucleotide sequences; these were translated into protein (amino acid) sequences using standard eukaryotic codon tables, and 3167 high-quality protein sequences were retained after quality filtering (S4 Table in S1 File). All subsequent protein-level processing—including feature extraction, data normalization, and model input—was performed using the identical pipeline as the internal dataset, ensuring consistency in model training and evaluation.

Overall performance (S5 Table in S1 File) analysis revealed that XGBoost achieved the highest accuracy (0.7729) and precision (0.7475), while CatBoost demonstrated optimal balance with peak recall (0.7666) and F1-score (0.7625). The Stacking model—though superior in internal validation—showed reduced efficacy externally (accuracy: 0.7240), indicating sensitivity to dataset distribution shifts. Class-specific evaluation (S6 Table in S1 File) further demonstrated that Anti-TT Ab achieved exceptionally strong classification performance under CatBoost, with accuracy of 0.98 and F1-score of 0.99, which can be attributed to the effective capture of conserved sequence motifs through our feature engineering. Anti-HIV-1 Ab maintained robust performance with an accuracy of 0.90, while Anti-FLU Ab exhibited notably lower accuracy, ranging from 0.52 to 0.57, consistent with the antigenic diversity characteristic of influenza. Classes with limited representation showed constrained performance, highlighting the dependence on sample size.

Critically, our models outperformed Wang et al.’s original approach on their native dataset, with XGBoost exceeding their Stacking accuracy by 6.0% and CatBoost delivering a notable relative F1-score improvement of approximately 38% for Anti-TT Ab. These results validate our feature engineering’s cross-dataset robustness while highlighting expected performance variations due to biological heterogeneity.

### Feature contributions for antigen-specific antibody prediction

**Fig 2:**
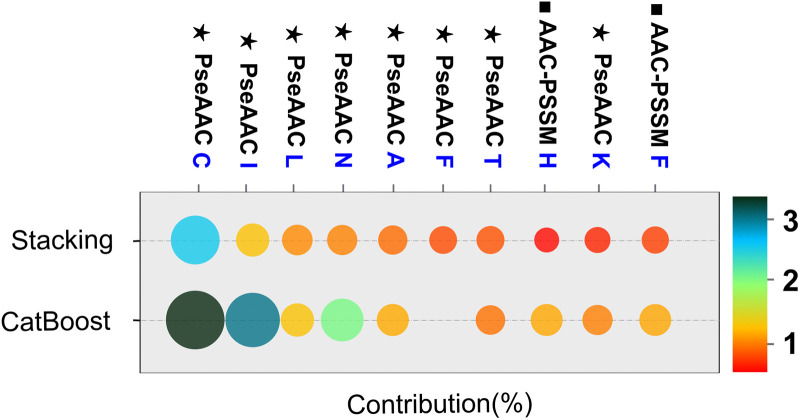
SHAP-based comparison of key feature contributions in the Stacking and CatBoost models. The horizontal axis represents the percentage contribution of each feature. Bubble size indicates the magnitude of feature importance, and color intensity reflects relative contribution strength. PseAAC and AAC-PSSM features are shown, highlighting the dominant role of sequence composition and evolutionary information.

To elucidate the factors contributing to antibody specificity classification, we employed the SHAP framework to quantify feature importance across three predictive models: CatBoost, Stacking, and Transformer (S4 Fig in S1 File). Subsequent analysis identified several features exhibiting significant influence across these models (Fig 2). SHAP analysis revealed that amino acid composition features derived from PseAAC (Pseudo Amino Acid Composition) and AAC-PSSM (Amino Acid Composition–Position-Specific Scoring Matrix) were predominant contributors to model outputs, suggesting their importance in distinguishing among labeled antibody classes. Notably, PseAAC features, particularly cysteine (C) and isoleucine (I), showed substantial contributions, with SHAP values of 2.86% and 1.10% in the Stacking model, and 3.97% and 3.49% in the CatBoost model, respectively. AAC-PSSM features, including histidine (H), phenylalanine (F), and glycine (G), also exhibited considerable influence. Phenylalanine (AAC-PSSM F) demonstrated consistent importance across models, with SHAP value contributions of 0.43% in Stacking, 0.12% in Transformer, and 0.95% in CatBoost. These residues possess physicochemical characteristics commonly consistent with known physicochemical properties relevant to protein interactions, including hydrophobic interactions, electrostatic adaptability, and conformational flexibility. Although region-specific annotations were not explicitly incorporated into feature extraction, the SHAP-identified patterns are consistent with previously reported sequence and structural characteristics of antibodies. Collectively, these findings suggest that the models capture informative patterns in the dataset rather than arbitrary compositional biases. Importantly, ambiguous residues were normalized during preprocessing. Although such normalization represents a practical compromise, the extremely low frequency of ambiguous residues indicates that the observed importance of amino acids such as alanine is unlikely to arise from artificial substitution effects. Caution is nevertheless warranted in interpreting this result, and improved encoding strategies will be explored in future work to address this limitation. Moreover, physicochemical properties and structural features, such as hydrophobicity (’ASC920101.G2’) and secondary structure propensities (’secondarystruct.G2’), contributed to model outputs, indicating their relevance in differentiating antibody classes. These findings collectively underscore the pivotal role of amino acid composition and evolutionary sequence information in driving the predictive power of the three models, elucidating how specific biological characteristics modulate the likelihood of accurate antigen classification.

**Fig 3:**
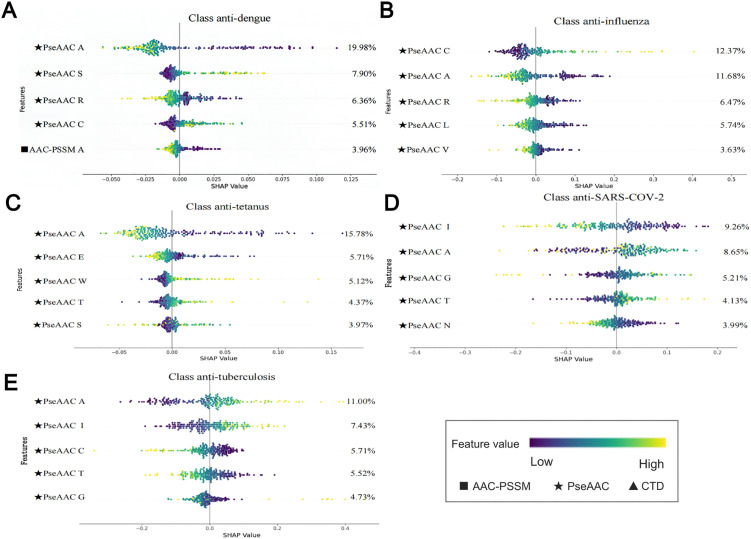
The top five most important features for each of the five antibody classes. Each class is shown in a separate subplot: anti-dengue antibodies (A), anti-influenza antibodies (B), anti-tetanus antibodies (C), anti-SARS-CoV-2 antibodies (D), and anti-tuberculosis antibodies (E). The dots on the right side of each subplot represent the SHAP values for individual samples.

Further dissection of the SHAP analysis revealed the top five contributing features for each antibody class (Fig 3). For anti-dengue (Fig 3A), anti-tetanus (Fig 3C), and anti-tuberculosis (Fig 3E) antibodies, PseAAC A emerged as a dominant factor, contributing 19.98%, 11.68%, and 11.00% to model outputs, respectively. In contrast, PseAAC C was the primary contributor (12.37%) for anti-influenza antibodies (Fig 3B), while PseAAC I played a leading role (9.26%) for anti-SARS-CoV-2 antibodies (Fig 3D).

## Discussion

Data-driven analysis of antibody specificity from sequence data represents an important direction in computational immunology and biotherapeutic development. Our study formulates this problem as a supervised classification task based on curated datasets, providing a practical framework for exploring sequence–function relationships. To this end, we implemented a comprehensive computational framework integrating multiple machine learning strategies, including individual classifiers, ensemble-learning techniques, and a feature-based Transformer architecture. These models are designed to capture complementary patterns in antibody sequence data associated with labeled specificity classes. Through systematic evaluation, we assessed their performance across five clinically relevant antibody categories, and applied SHAP analysis to identify sequence-derived features that contribute most strongly to classification outcomes.

A variety of antigen-antibody binding-based recognition models have been developed in biomedical research, such as A2binder [34], AntiFormer [35], AlphaFold [36,37], which have demonstrated strong capabilities in predicting and evaluating antigen-antibody binding affinities. It is worth noting that modern tools like AlphaFold [38] and ESMFold [39] can reliably predict antigen structures from sequence, their accuracy for transient antibody-antigen complexes remains limited [40]. In this context, our sequence-based framework provides a complementary strategy when structural data are unavailable, focusing on classification using physicochemical, sequence-order, and evolutionary features derived from antibody sequences.

Our comprehensive analysis revealed nuanced performance across different models and antibody classes. Among individual models, CatBoost demonstrated superior performance with an accuracy of 0.7713, outperforming other classifiers. The Stacking ensemble strategy marginally improved recognition performance, achieving an accuracy of 0.7803 and an F1 score of 0.7802, underscoring the potential of integrating diverse learning algorithms. Notably, our models exhibited varying efficacy in classifying different antigen-specific antibodies. Antibodies against Mycobacterium tuberculosis, SARS-CoV-2, and influenza virus were consistently well-classified across models, with accuracy ranging from 0.76 to 0.86. This superior performance may be attributed to the distinct evolutionary pressures exerted by these pathogens, resulting in more conserved epitope recognition patterns within their respective antibody repertoires. The heightened immunogenicity and unique structural features of these pathogens likely contribute to more distinguishable antibody signatures. Conversely, the classification of dengue virus-specific antibodies proved challenging, with the Transformer model yielding the highest yet suboptimal performance (accuracy: 0.27; F1-score: 0.26). This suboptimal performance is likely attributable to two key factors: (1) the absence of serotype annotation (DENV-1 to DENV-4) in the NCBI dataset, leading to aggregation of serotype-specific and cross-reactive antibodies under a single label; (2) extensive cross-reactivity among dengue serotypes and related flaviviruses, which blurs distinctive sequence features in antibody heavy chains. Future studies incorporating serotype-resolved datasets and refined labeling strategies may improve classification accuracy and enable more precise modeling of dengue-specific antibody repertoires [41].

Most models in this study yielded comparable overall accuracy at approximately 77%, with little global performance divergence under the current experimental setup, a pattern driven by three core factors. First, the modest dataset of 1111 sequences for five-class multi-class classification constrains the discriminative capacity of complex models. Second, all models were trained on the identical 81-dimensional engineered feature space, which already encodes substantial sequence-derived information—this means the performance ceiling is largely determined by feature informativeness rather than algorithmic complexity. Third, intrinsic feature overlap between antibody classes, especially for anti-dengue antibodies, reduces sequence-feature level separability and caps achievable accuracy across all classifiers. Notably, similar overall accuracy does not equate to identical class-level performance: marked differences in precision and recall exist for specific antibody classes, such as the Stacking model’s superior performance for anti-SARS-CoV-2 antibodies, demonstrating that models capture distinct decision boundaries despite matching global metrics. This aligns with findings from related antibody and protein-sequence classification studies, where diverse machine learning algorithms generate comparable global metrics when applied to the same handcrafted feature space.

To investigate factors contributing to antibody specificity classification, we used SHAP analysis to interpret how sequence‑derived biological features contribute to classification. The results revealed that both evolutionary (AAC‑PSSM) and physicochemical (PseAAC) descriptors capture patterns critical to antigen specificity (Fig 2). Among these, cysteine (C) consistently exhibited high SHAP contributions across models. However, its predictive influence should be interpreted cautiously, as cysteine residues in VH domains mainly form conserved intrachain disulfide bonds rather than direct antigen‑contact residues. Therefore, their high SHAP importance likely reflects structural stability that indirectly supports proper paratope conformation rather than a true specificity determinant. Isoleucine (I), a hydrophobic amino acid, contributes to the core stability of proteins, which is vital for their folding and activity [42]. The AAC-PSSM-based scores for Histidine (H) and Phenylalanine (F) are indicative of their critical roles in protein structure and function. Histidine, renowned for its capacity to engage in hydrogen bonding and proton transfer mechanisms [43], is integral to the stability and catalytic proficiency of protein. Meanwhile, Phenylalanine, characterized by its hydrophobic side chain, is instrumental in establishing the protein’s hydrophobic core, thereby bolstering its overall folding and stability [44,45]. These attributes are seminal to protein-protein interactions and molecular recognition processes, which may influence protein structural properties. These features help the model identify functional regions, assess structural stability, and predict protein anomalies, with SHAP values emphasizing their contribution to prediction accuracy.

The arrangement and chemical properties of amino acids are known to influence antibody structure and antigen-binding behavior. In our analysis, antibodies related to SARS-CoV-2 and Mycobacterium tuberculosis showed relatively strong classification performance, which may be associated with the contribution of amino acids such as alanine (Ala), isoleucine (Ile), and glycine (Gly) within their sequences. Previous studies have shown that alanine, due to its small hydrophobic methyl side chain, is often involved in stabilizing protein interfaces, including VH–VL interactions, and may contribute to maintaining structural integrity in antibody variable domains [46–48]. Isoleucine, a branched-chain hydrophobic residue, has been reported to play a role in maintaining protein core stability and is frequently observed in hypervariable regions, where it may support local structural organization [49–51]. Glycine, owing to its lack of a side chain, confers conformational flexibility and is commonly enriched in loop regions such as CDR-H3, which are associated with structural adaptability [52,53]. In the context of our study, the importance of these residues identified by SHAP analysis is consistent with their reported physicochemical and structural roles. These properties may contribute to sequence-derived feature patterns that facilitate discrimination between antibody classes.

Building upon our findings, it is crucial to acknowledge the limitations of our study to contextualize our results and guide future research directions. First, data composition and antigen heterogeneity constrain model generalizability. Certain antigen classes exhibit inherent labeling biases. For example, anti-tetanus antibodies target tetanus toxoid rather than Clostridium tetani itself, and influenza antibodies are influenced by antigenic drift as well as vaccination-induced immune responses. Additionally, dengue antibody data lack consistent serotype annotation and contain occasional incomplete sequence records, all of which may introduce antigen-specific bias and limit generalization across subtypes or evolving variants. Second, our modeling framework primarily relies on global sequence descriptors (AAC-PSSM, PseAAC, and CTD), which capture evolutionary and physicochemical properties but do not directly localize feature importance to defined complementarity-determining region (CDR) domains. As a result, SHAP interpretations remain feature-level rather than paratope-focused. Region-specific features—particularly those derived from CDR-H3—may provide more direct mechanistic insight. CDR-H3 length is closely associated with antigen-binding diversity and structural flexibility, while aromatic enrichment, localized hydrophobicity, and net charge at physiological pH can influence antigen recognition through interface packing and electrostatic steering effects. Because these localized determinants are implicitly embedded but not explicitly encoded, antigen-specific variability may be partially diluted by conserved framework regions. This representation-level limitation may contribute to the observed performance ceiling and suggests that incorporating CDR-focused descriptors could enhance interpretability and discriminative resolution in future models. Third, the current sequence representation does not explicitly incorporate structural or three-dimensional binding information. While manual feature engineering improves interpretability and robustness under limited data conditions, it may miss higher-order structural determinants of antigen recognition. Integrating structural modeling or pretrained protein language models (e.g., ESM-2 [54,55] or ProtBERT [56]) may help capture implicit structural-functional patterns beyond handcrafted descriptors. Fourth, class imbalance—particularly the relatively limited anti-dengue samples—may affect classification stability. Although preliminary resampling strategies were explored, maintaining biological authenticity and avoiding feature distortion remain challenges. Future work may explore data augmentation, refined resampling strategies, or transfer learning approaches to mitigate imbalance-related bias. Fifth, dataset heterogeneity presents potential confounding factors. Because antibody sequences were aggregated from multiple species and independent studies, models may inadvertently capture species-specific germline signatures or VH/VJ usage patterns rather than purely antigen-driven specificity. Future analyses should incorporate species-stratified evaluation, VH/VJ usage statistics, and CDR-H3 distribution comparisons to better disentangle antigen specificity from lineage or study-origin effects. A further data-related consideration is the moderate 40% sequence identity threshold applied to reduce redundancy: this cutoff was selected to balance the removal of closely related homologs and preservation of functional sequence diversity, yet global identity filtering cannot fully eliminate structural similarity across samples, particularly within the conserved framework regions of antibody heavy chains. Moreover, our reliance on a single public dataset consortium limits real-world applicability, as sensitive antibody sequence data is typically siloed across institutions. To bridge this gap and align with clinical discovery workflows, future work will implement privacy-preserving multi-institutional training frameworks, such as adaptive federated learning (FL) with differential privacy (DP) guarantees [57], enabling collaborative model development without centralizing sensitive data. To further ensure secure, compliant cross-site collaboration, we will integrate blockchain-based access control frameworks [58], which provide tamper-proof audit trails and fine-grained permission management—essential safeguards for adhering to regulations like GDPR and fostering trust in multi-stakeholder research consortia. Beyond data and feature engineering constraints, our heavy-chain-only modeling framework overlooks light-chain contributions to antigen binding; while heavy-chain screening is valuable for large-scale candidate prioritization, functional validation of antibody candidates ultimately requires paired heavy- and light-chain expression and experimental characterization. Finally, model architecture and training constraints limited the full potential of our deep learning approaches. While our feature-based Transformer outperformed traditional machine learning methods, its expressiveness was constrained by the small, imbalanced nature of our dataset—a pervasive challenge in antibody informatics. This design prioritizes interpretability via biologically informed feature engineering (encoding evolutionary, physicochemical, and structural properties) over the raw sequence representation learning of large protein language models (e.g., ESM-2, ProBERT), which require extensive pretraining and large downstream datasets to excel.

## Conclusions

In conclusion, this study presents a computational framework for analyzing antibody specificity from sequence data. By leveraging a diverse set of machine-learning approaches, we demonstrate the feasibility of classifying antibodies across five clinically relevant categories within curated datasets. Our results highlight the potential of data-driven methods in antibody research and suggest that sequence-derived features capture informative patterns associated with antibody specificity.

## Supporting information

S1 File All supporting information tables and figures, including model performance metrics, independent dataset distributions, and visualization results.
(DOCX)
