# Supplementary material for: Computational models for the classification of antibody specificity using heavy chain features
Source: PLoS One. 2026 May 20;21(5):e0349143. doi: 10.1371/journal.pone.0349143 (PMC13189292; doi:10.1371/journal.pone.0349143)

Supplementary Tables for

**Computational Models for the Classification of Antibody Specificity Using Heavy Chain Features**

Jia Lin^1^, Jiaqi Chen^1^, Linxuan Wan^5^, Weinan He^1^, Yuxin Zhu^1^, Mu Qiao^6^, Fancun Meng^1^, Di Lin^2^, Yan Che^2^, Zicheng Cao^1,2,3,4^

Corresponding to: Dr. Zicheng Cao, School of Public Health, Shantou University, Shantou, P.R. China.

Email: [zichengcao@stu.edu.cn](mailto:zichengcao@stu.edu.cn)

**Affiliation:**

1. School of Public Health, Shantou University, Shantou 515041, P.R. China

2. Engineering Research Center for Big Data Application in Private Health Medicine of Fujian Universities, Putian University, Putian 351100, P.R. China

3. School of Public Health (Shenzhen), Shenzhen Campus of Sun Yat-sen University, Shenzhen 518107, P.R. China

4. Shenzhen Key Laboratory of Pathogenic Microbes & Biosafety, Shenzhen Campus of Sun Yat-sen University, Shenzhen 518107, P.R. China

5. School of Mathematics and Computer Science, Shantou University, Shantou 515041, P.R. China

6. Local Government Development Research Institute of Shantou University, Law School of Shantou University, Shantou 515041, P.R. China

**S1 Table.** **Performance of stacking model on the testing dataset.**

| **Classifier** | **Accuracy** | **F1score** | **Precision** | **recall** |
| --- | --- | --- | --- | --- |
| **Stacking** | **0.7803** | **0.7802** | **0.7843** | **0.7803** |
| LR | 0.6910 | 0.6870 | 0.6950 | 0.6910 |
| RFC | 0.7310 | 0.7130 | 0.7180 | 0.7310 |
| SVM | 0.7490 | 0.7550 | 0.7740 | 0.7490 |
| KNN | 0.7040 | 0.7080 | 0.7210 | 0.7040 |

**S2 Table.** **Performance of model on the training dataset.**

| **Classifier** | **Accuracy** | **F1-score** | **Precision** | **Recall** |
| --- | --- | --- | --- | --- |
| **CatBoost** | **0.7568** | **0.7557** | **0.7588** | **0.7568** |
| XGBoost | 0.7444 | 0.7390 | 0.7475 | 0.7443 |
| LGBM | 0.7422 | 0.7369 | 0.7401 | 0.7422 |
| AdaBoost | 0.7230 | 0.7127 | 0.7176 | 0.7230 |
| RF | 0.6937 | 0.6767 | 0.6781 | 0.6937 |
| **Stacking** | **0.8590** | **0.8581** | **0.8587** | **0.8590** |
| **Transformer** | **0.6800** | **0.6780** | **0.6650** | **0.6920** |

**S3 Table.** **Macro-Averaged AUROC and AUPRC for Different Classifiers.**

| **Classifier** | **Macro-Averaged AUROC** | **Macro-Averaged AUPRC** |
| --- | --- | --- |
| **CatBoost** | **0.9480** | **0.7582** |
| XGBoost | 0.8701 | 0.6512 |
| LGBM | 0.9247 | 0.7089 |
| AdaBoost | 0.9250 | 0.7114 |
| RF | 0.9195 | 0.7015 |

**S4 Table.** **Distribution of antigen-specific antibodies in the external validation dataset.**

| **Label** | **Class** | **Number of Sequence Entries** |
| --- | --- | --- |
| 1 | Anti-HIV-1 Ab | 1287 |
| 2 | Anti-FLU Ab | 700 |
| 3 | Anti-PPS Ab | 245 |
| 4 | ACPA | 496 |
| 5 | Anti-TT Ab | 276 |
| 6 | Anti-HBV Ab | 162 |

**S5 Table.** **Performance of various classifiers on external validation dataset.**

| **Classifier** | **Accuracy** | **F1-score** | **Precision** | **Recall** |
| --- | --- | --- | --- | --- |
| **CatBoost** | **0.7666** | **0.7625** | **0.7750** | **0.7666** |
| XGBoost | 0.7729 | 0.7390 | 0.7475 | 0.7443 |
| LGBM | 0.7713 | 0.7369 | 0.7401 | 0.7422 |
| AdaBoost | 0.7114 | 0.7030 | 0.7197 | 0.7114 |
| RF | 0.7534 | 0.7383 | 0.7788 | 0.7534 |
| **Stacking** | **0.7240** | **0.7198** | **0.7226** | **0.7240** |
| **Transformer** | **0.7114** | **0.7129** | **0.7195** | **0.7114** |

**S6 Table.** **Classification performance of different classifiers for antibody specificity on the external validation dataset.**

| Class | Accuracy | | | F1-score | | | Precision | | | Recall | | |
| --- | --- | --- | --- | --- | --- | --- | --- | --- | --- | --- | --- | --- |
|  | **C** | **T** | **S** | **C** | **T** | **S** | **C** | **T** | **S** | **C** | **T** | **S** |
| Anti-HIV-1 Ab | 0.90 | 0.81 | 0.83 | 0.81 | 0.81 | 0.79 | 0.74 | 0.82 | 0.75 | 0.90 | 0.81 | 0.83 |
| Anti-FLU Ab | 0.57 | 0.53 | 0.52 | 0.64 | 0.57 | 0.54 | 0.72 | 0.61 | 0.57 | 0.57 | 0.53 | 0.52 |
| Anti-PPS Ab | 0.79 | 0.83 | 0.76 | 0.88 | 0.73 | 0.82 | 1.00 | 0.66 | 0.90 | 0.79 | 0.83 | 0.76 |
| ACPA | 0.63 | 0.61 | 0.57 | 0.64 | 0.56 | 0.59 | 0.64 | 0.51 | 0.61 | 0.63 | 0.61 | 0.57 |
| Anti-TT Ab | 0.98 | 0.80 | 0.96 | 0.99 | 0.87 | 0.92 | 1.00 | 0.96 | 0.89 | 0.98 | 0.80 | 0.96 |
| Anti-HBV Ab | 0.64 | 0.58 | 0.58 | 0.78 | 0.58 | 0.71 | 1.00 | 0.58 | 0.90 | 0.64 | 0.58 | 0.58 |

Note: C represents CatBoost, T represents Transformer, and S represents Stacking.

**S1 Fig.** **Schematic overview of the data preprocessing, feature extraction, and validation workflow.** Raw antibody sequences were retrieved from NCBI and subjected to modification filtering, ambiguous residue removal, and redundancy reduction using CD-HIT (40% identity threshold), resulting in 1,111 non-redundant sequences. An 81-dimensional feature vector was extracted for each sequence using AAC-PSSM, PseAAC, and CTD descriptors. The dataset was divided into an 80% training set (n = 888) and a 20% independent internal test set (n = 223). Within the training set, five-fold cross-validation was applied, and out-of-fold (OOF) predictions were used to train the stacking meta-learner. Final model performance was evaluated on the internal test set and further validated using the independent Wang dataset.

**
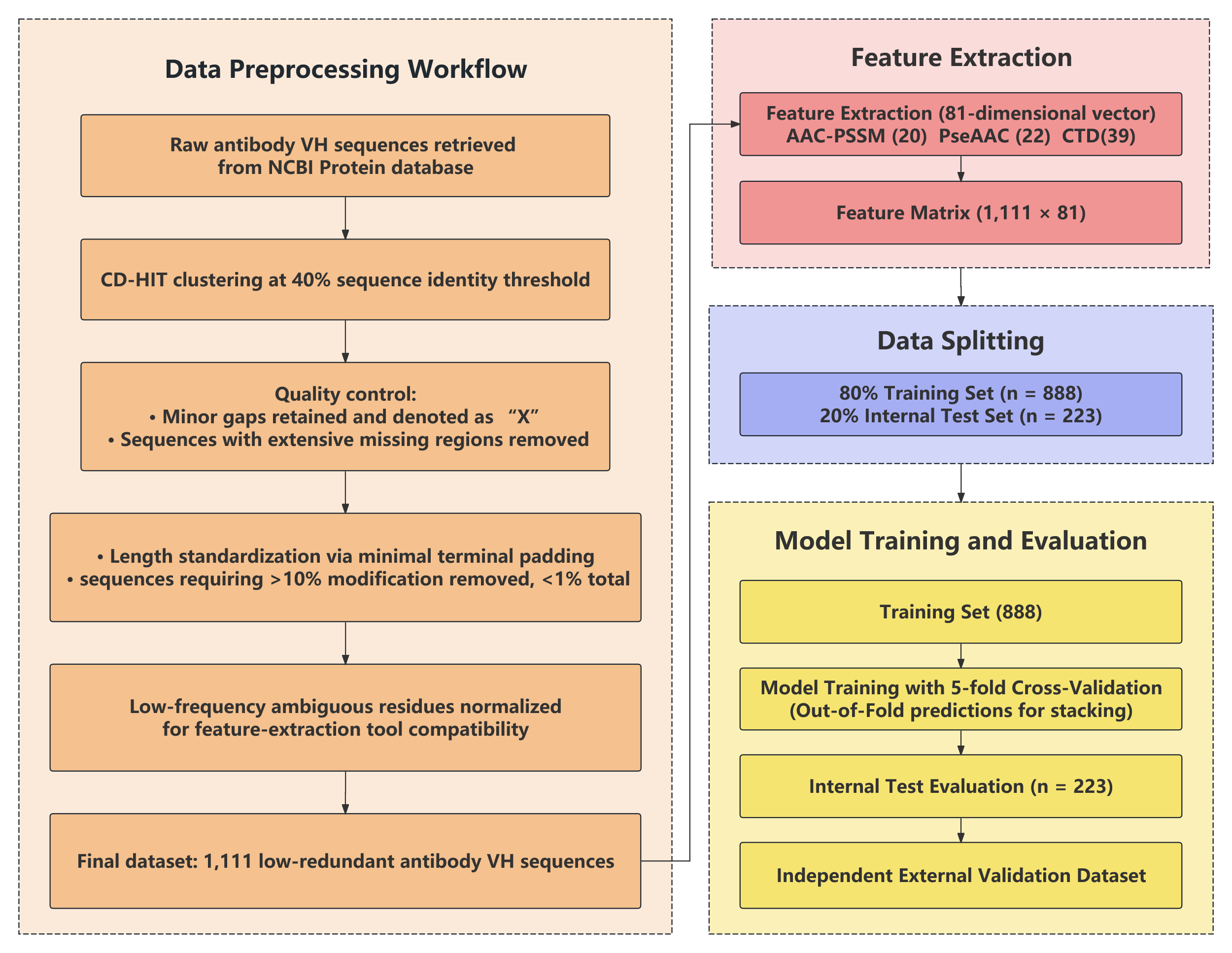
**

**S2 Fig. Confusion Matrices for (A) CatBoost, (B) Stacking, and (C) Transformer Models.**

**
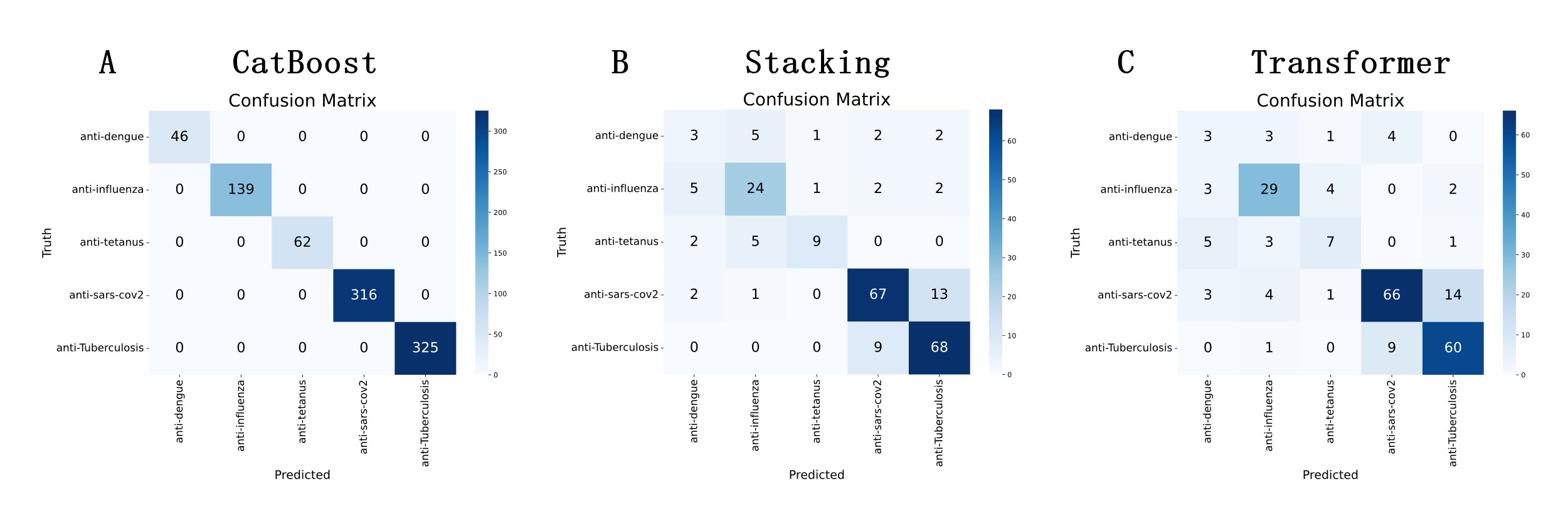
**

**S3 Fig. (A) Change curve of test set and training set accuracy during training. (B)Change curve of loss function between test set and training set during training.**


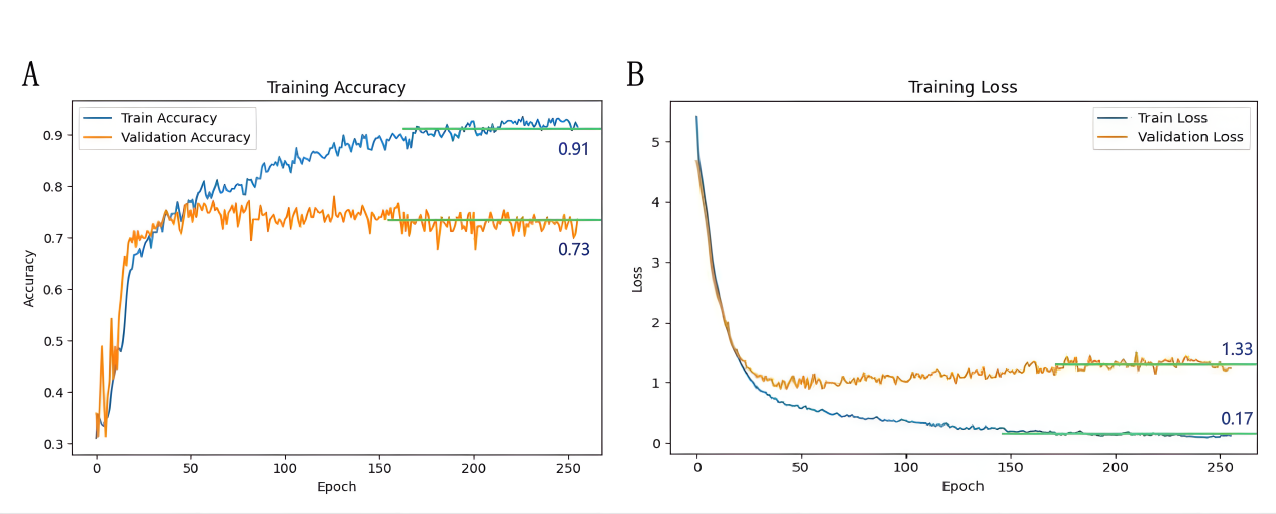


**S4 Fig.** **Top 30 Features for (A) CatBoost, (B) Stacking, and (C) Transformer Models.**

**
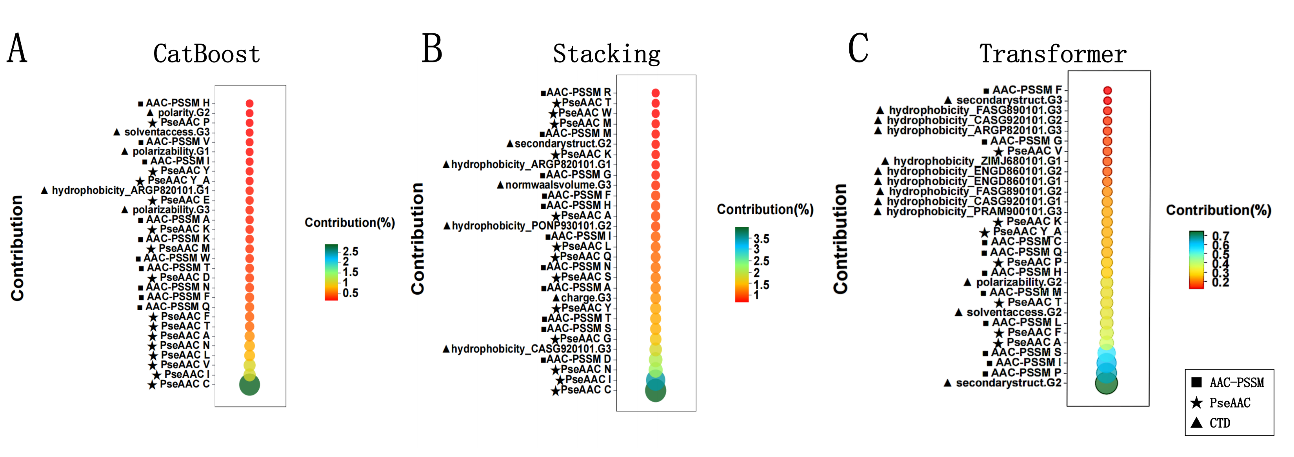
**

**S5 Fig. ROC Curves for Multi-Class Classification: (A) CatBoost, (B) XGBoost, (C) LGBM, (D) AdaBoost, (E) RF.**


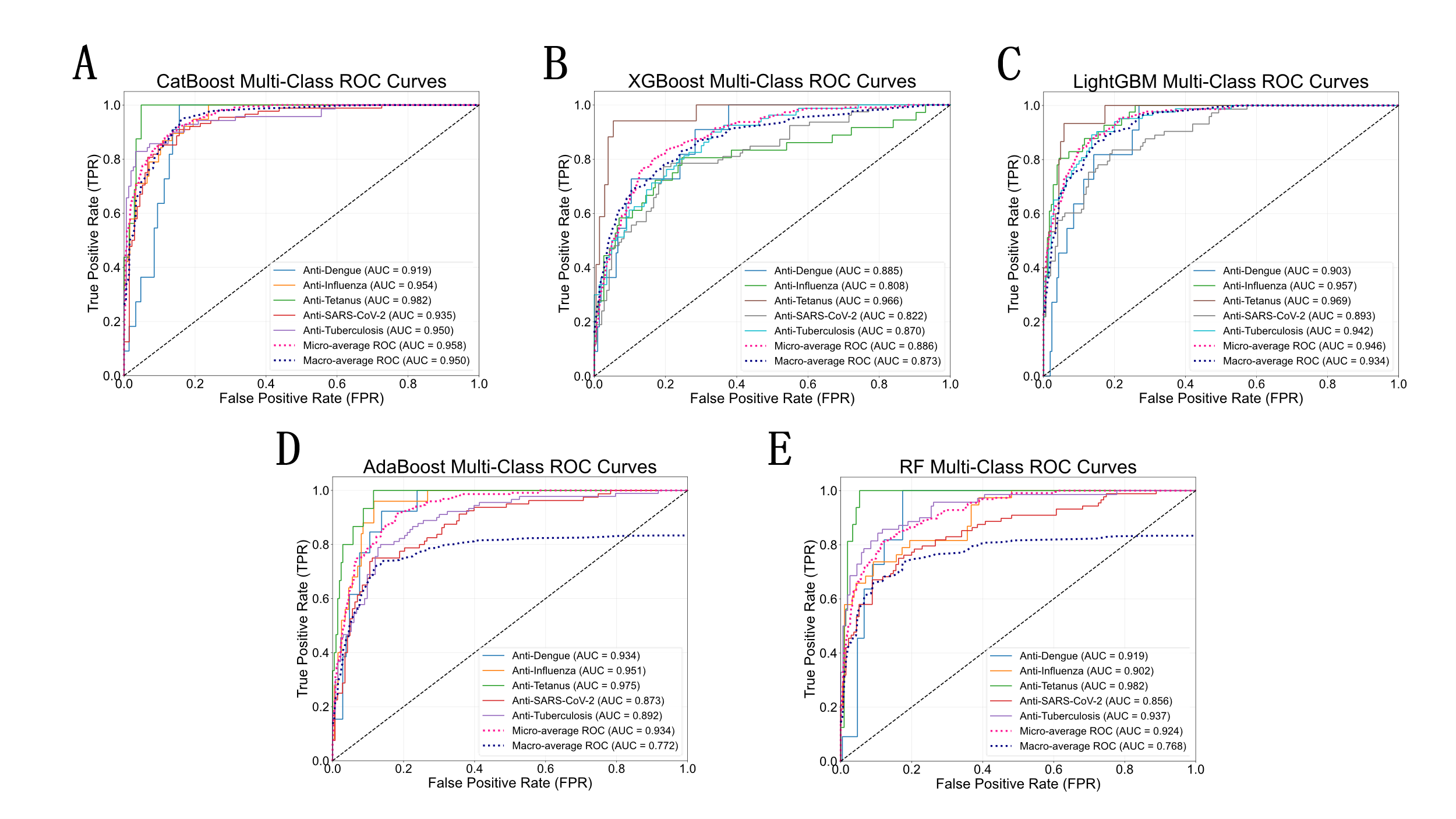

Supplement: S1 File — All supporting information tables and figures, including model performance metrics, independent dataset distributions, and visualization results. (DOCX) [file pone.0349143.s001.docx]
